# Task completion time: Objective tool for assessment of technical skills in laparoscopic simulator for urology trainees

**DOI:** 10.4103/0970-1591.38601

**Published:** 2008

**Authors:** Shashi K. Mishra, A. Ganpule, A. Kurien, V. Muthu, Mahesh R. Desai

**Affiliations:** Department of Urology and Nephrology, Muljibhai Patel Society for Research in Nephrourology, Muljibhai Patel Urological Hospital, Nadiad - 387 001, Gujarat, India

**Keywords:** Beetle universal, endotrainer, laparoscopy, simulation, task completion time

## Abstract

**Context:**

Laparoscopic surgical simulation is a valuable training tool for urology trainees.

**Aims:**

We assessed the validity of task completion time (TCT) as an objective tool for practicing and acquiring technical skills in a simulated laparoscopy environment.

**Materials and Methods:**

Fifteen participants comprising postgraduate urology trainees from first to third year (n = 12), urology fellow (n = 1) and consultants (n = 2) underwent basic laparoscopic training on the “Beetle Universal” endotrainer. Training included 10 attempts each comprising four tasks; placing a ball in a cup (Task 1), threading five rings (Task 2), threading five balls (Task 3) and tying a suture (Task 4). Individual task (IT) time was measured. The TCT was defined as sum of IT time for a single attempt.

**Statistical Analysis Used:**

Statistical analysis was done by Pearson's correlation coefficient and student's t test using SPSS software 10.

**Results:**

The average TCT for the first attempt to complete the four tasks by the participants was 76.5 ± 13.0 min (range 38 to 92.5, skew −1.8), compared to the 10^th^ attempt 33 ± 4.23 min (range 25 to 38.5, skew −0.5). There was statistically significant correlation (r = mean −0.91, range −0.97 to −.83, skew −0.5), (*P* = < 0.001) between the number of attempts and decreasing TCT for all participants. Correlation decreased when TCT between the sixth to 10^th^ attempt was compared (r = mean −0.67, range −0.99 to 0.76).

**Conclusions:**

The TCT is practical, easy and a valid objective tool for assessing acquired technical skills of urology trainees in a laparoscopic simulated environment.

## INTRODUCTION

Various ethical, medico-legal and health economy demands have made training in laparoscopic urological surgery challenging. There remains a lack of consensus on the optimal training program.[1] The optimal training program should be based on individual institution, resources, economics, mentor availability and patient load.[1] Teaching of basic surgical skills is both feasible and advantageous using simulation.[2] Objective assessment of simulation performance skills is integral to the success of the concept. Without performance validity, simulation training would not acquire credibility and value.[3] We assessed the task completion time (TCT) as a simple objective tool for both acquiring and practicing technical skills in a simulated laparoscopy environment.

## MATERIALS AND METHODS

**Figure 1:**
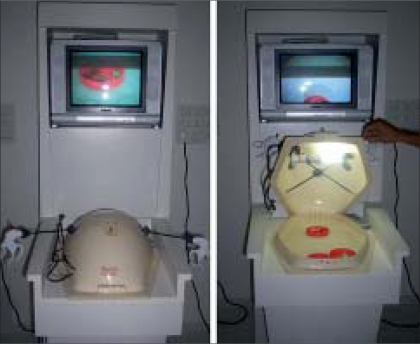
Beetle universal (ethicon endo-surgery)

**Figure 2:**
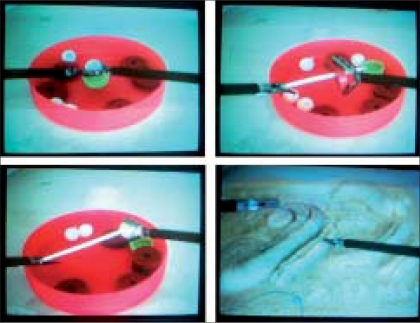
Tasks view on screen, Left upper corner (Task 1), Right upper corner (Task 2), Left lower corner (Task 3), Right lower corner (Task 4)

Fifteen participants comprising urology residents from the first to third year of training (n = 12), urology fellow (n = 1) and consultants (n = 2) underwent basic laparoscopic training on the “Beetle Universal, Ethicon Endo-surgery” endotrainer [Figure 1]. None of the participants had experience in live laparoscopic procedures. Training involved 10 attempts each comprising four tasks to be performed sequentially on the endotrainer. The tasks involved: placing a ball in a cup (Task 1), threading five rings (Task 2), threading five balls (Task 3) and tying a suture (Task 4). An attempt was defined as performing the four tasks sequentially. Instruments were a laparoscopic grasper (Endopath, Ethicon Endo-surgery) for the left hand and a laparoscopic Maryland (Endopath, Ethicon Endo-surgery) for the right hand. The first task was to grasp a cup with the left hand, then pick up a ball with the right hand and place it into the cup. By doing this, the trainee gained skills in hand-eye coordination for the movement of the instrument in the 2-D plane. The second and third tasks involved 3-D manipulation of the laparoscopic instruments. The trainee acquired coordination of the movement by picking up a total of five rings and balls (Tasks 3 and 4) with the Maryland and threading them onto a rod held by the laparoscopic grasper [Figure 2]. The fourth task was to tie a suture on a porous mattress with the help of both lap instruments [Figure 2]. Individual task (IT) time was measured during each attempt. The TCT was defined as sum of IT time to perform all four tasks in a single attempt. Each participant performed 10 attempts with or without interruption over a period of one to five days. Data was analyzed after the end of the 10^th^ attempt.

Statistical analysis was done by Pearson's correlation coefficient and student's paired t test using SPSS software 10. Values were considered statistically significant for *P* values less than 0.01.

## RESULTS

**Figure 3:**
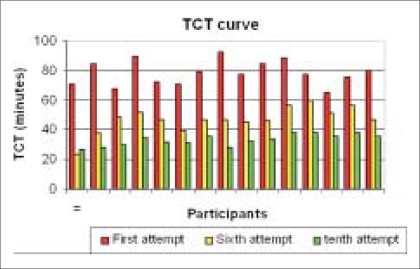
TCT comparison at first, sixth and 10^th^ attempt

**Figure 4:**
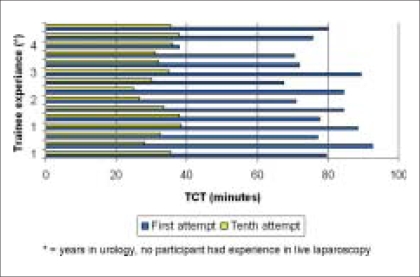
TCT comparison with trainee experience

The average TCT for first attempt by the participants was 76.5 ± 13.0 min (range 38 to 92.5, skew −1.8), compared to the 10^th^ attempt 33 ± 4.2 minutes (range 25 to 38.5, skew −0.5) [Table 1]. There was statistically significant correlation (r = mean −0.91, range −0.97 to −.83, skew −0.5), (*P* = < 0.001) between the number of attempts and decreasing TCT time for all participants. Correlation decreased when TCT between the sixth to 10^th^ attempt was compared (r = mean −0.67, range −0.99 to 0.76) [Figure 3]. Improvement in TCT did not correlate with trainee experience (r = −0.4 at first attempt and 0.28 at 10^th^ attempt) [Figure 4]. Although increasing attempts decreased the IT time for all the four tasks, significant difference was demonstrated for Tasks 2 and 3 (*P* value 100% significant for all participants *vs.* 6% and 13% for Tasks 1 and 4).

**Table 1:** Results

	First attempt	Sixth attempt	10^th^ attempt
Mean	76.5	46.9	33
SD	13.01	8.80	4.23
Skewness	−1.85	−1.21	−0.50
Kurtosis	5.19	2.69	−0.72
Median	77.5	47	33.5

Pearson's correlation coefficient (r) for first to 10^th^ attempt = mean −0.91(range −0.97 to −0.83, *p* = <0.001*. Pearson's correlation coefficient (r) for sixth to 10^th^ attempt = mean −0.67 (range −0.99 to 0.76)

Significant skewness and kurtosis clearly indicate that data are not normal. Normal distributions produce a kurtosis statistic of about zero. Positive kurtosis indicates a “peaked” distribution and negative kurtosis indicates a “flat” distribution. The existence of flat or peaked distributions as indicated by the kurtosis is important to us as a marker. It indicates violations of the assumption of normality that underlies many of the other statistics like correlation coefficients, t-tests, etc. used to study the validity of a test.

At the end of the 10^th^ attempt by trainees in our study, the kurtosis was −0.72 suggesting the near normal distribution of results and hence more validity.

A skewed distribution may actually be a desirable outcome on a criterion-referenced test. For example, a near zero skewed distribution (–0.50 in this study) with all trainees scoring very high on the 10^th^ attempt may simply indicate that the hand-eye coordination and cognitive ability improved well. This was especially true because the trainees had previously scored poorly in a negatively skewed distribution (–1.85 at first and –1.21 at sixth attempt) at the beginning of the test. In fact, the difference between the more negatively skewed distribution at the first attempt and the near zero skewed distribution at the end of the 10^th^ attempt was an indication of how much the trainees learned during the course of the study.

## DISCUSSION

A revolution has occurred in surgical education, as a result of the availability of surgical simulators for the training and assessment of technical skills. This new innovation is due to a combination of simulators, curriculum-based training, validation of objective assessment and policy (criterion-based benchmarks) to deliver high-level proficiency-based training.[4] This gives us an insight into the fundamental shift in the way budding urologists will be trained in the future. Technical competence in laparoscopic surgery depends on the development of basic abilities and skills peculiar to the technique. Operating on 3-dimensional objects from a 2-dimensional image projected on a video screen is the basic cognitive ability required in laparoscopy. This involves a visual depth perception component as well as a psychomotor component. The basic principles of mechanical simulators have not changed during the last decade, however, the types of have been constantly upgraded over the years.[5]

A series of tasks are integrated to simulate a surgical procedure.[6] Several metrics for objective assessment of acquiring skills have been proposed. The McGill Inanimate System for Training and Evaluation of Laparoscopic Skills (MISTELS) is the physical simulator incorporated by the Society of American Gastrointestinal and Endoscopic Surgeons (SAGES) in their fundamentals of laparoscopic surgery (FLS) program.[7] Skills assessment in the McGill system[8] is based on time to completion with penalty points deducted for imprecision.[9] The MISTELS metrics have been shown to have high interrater and test-retest reliability and to correlate with skill in animal surgery.[2] This system has also been validated for urology trainees.[9] However, the system does not measure qualitative details of laparoscopic movements. Such a refinement is now available in the Blue DRAGON Markov Model system developed by Rosen and associates,[10] as well as the Computer enhanced laparoscopy training system (CELTS) system developed by Stylopoulos.[11]

The main disadvantage of the above simulation devices is their inaccessibility in Indian institutions. The endotrainer should be easily available, user-friendly and above all cost-effective. Currently two endotrainers are available for training in India. The “Beetle Universal” trainer, a video-assisted device, indigenously developed, (design patent registered, Dr. U. S. Gadgill, Ethicon Endo-surgery) is readily available in India and meets all requirements of an endotrainer. This simulator is very similar to an inflated human abdomen and has multiple ports, helping the learner to do fulcrum movement and experience the visual depth orientation. The other BEST-IRIS simulator consists of an indigenously developed hardware to simulate a laparoscopic surgery setup prepared for the procedures on an operating table. This hardware runs on Microsoft Windows-based personal computer (PC) software.[12] In India, currently, trainee's acquisition of technical skill is either by subjective assessment by the trainee/trainer (Beetle Universal) or virtual reality technology (BEST-IRIS).

Once the minimum skills have been acquired for safe tissue handling, a mentor in live laparoscopy situations in animal models can guide the trainee. Both these procedures can be used as video feedback to facilitate confidence and competency, which can be achieved safely and quickly by most trainees. However, a validation study of the non-PC-based simulator “Beetle Universal” is required to affirm its appropriateness as a testing and certifying modality. Currently, there is no published data on validation studies of the “Beetle Universal” endotrainer. For application in any structured training program, an objective tool for measuring the level of acquisition of skills is required.

The advantage of using TCT as an objective tool is its easy application and validity. The performance is not biased by the variations in anatomy or physiologic response found in animals. The same test can be repeated in identical fashion at any location at any time. Second, the equipment is inexpensive, reusable and easy to set up quickly without an experienced staff.[13] As the number of attempts increased in this study, the TCT improved consistently for all participants. This may be due to improved hand-eye coordination. However, after a certain level of acquisition of skills, further improvement was slow. The TCT incorporates sum of IT time of four different simulator tasks, thereby giving construct validity. These four individual tasks may actually be directed at evaluating different cognitive ability giving TCT content validity.

The results showed that repetition of the tasks as listed above in the endotrainer had a positive effect on the 2D and 3D visual depth perception by the participants. As the total time spent on training increased, decreasing TCT reflected improvement in cognitive ability. Different participants had similar first and 10^th^ attempt TCT, irrespective of their experience in urology training as none of them had experience in laparoscopy. Overall improvement in the TCT was also similar between the participants' group, suggesting a learning curve in the basic cognitive ability required for laparoscopy. This also suggests that the learning curve is not dependent upon non-laparoscopic urological experience.

The delay between attempts for some participants during the study (one to five) days is a limitation of this study. This may give false high TCT at the end of the 10^th^ attempt. In addition, correlation with the clinical scenario was less. Further prospective validation and reliability (inter-observer) studies are needed to confirm the advantages of TCT as an objective tool.

## CONCLUSION

The TCT can easily be incorporated into the current training program for urology trainees in institutions using the indigenous “Beetle Universal” endotrainer. Number of attempts shows improvement in TCT suggesting a learning curve for laparoscopy in simulated environment. Once a certain level of proficiency is gained within the laparoscopy-simulated environment, trainees can be offered live laparoscopy situations.
